# Two new species of *Archaeopodagrion* (Odonata, Philogeniidae) from the western foothills of the Tropical Andes, with biological observations and distributional records

**DOI:** 10.3897/zookeys.1036.64230

**Published:** 2021-05-05

**Authors:** Vanessa Amaya-Vallejo, Cornelio Bota-Sierra, Rodolfo Novelo-Gutiérrez, Melissa Sánchez-Herrera

**Affiliations:** 1 Laboratorio de Zoología y Ecología Acuática, Universidad de los Andes, Bogotá DC, Colombia Universidad de los Andes Bogotá Colombia; 2 Grupo de investigación en Genética Evolutiva, Filogeografía y Ecología de Biodiversidad Neotropical, Universidad del Rosario, Bogotá DC, Colombia Universidad del Rosario Bogotá Colombia; 3 Red de Biodiversidad y Sistemática, Instituto de Ecología AC, Xalapa, Veracruz, México Red de Biodiversidad y Sistemática, Instituto de Ecología AC Xalapa Mexico; 4 Grupo de Entomología, Universidad de Antioquia, Medellín, Antioquia, Colombia Universidad de Antioquia Medellín Colombia

**Keywords:** Biodiversity hotspots, Colombia, damselfly, Ecuador, female, larva, male

## Abstract

Two new species of the damselfly genus *Archaeopodagrion*, *A. recurvatum***sp. nov.** and *A. mayi***sp. nov.**, are described from the confluence of the Tropical Andes and the Tumbes-Chocó-Magdalena biodiversity hotspots. Adults differ from the other known species in the shape of female posterior lobe of pronotum and male structures of cerci and paraprocts; the larva differs from other *Archaeopodagrion* species in the caudal lamellae structure and in the mandibular formula. The two new species are diagnosed, a morphological key to all known males and females in the genus is provided, and geographical distributions are updated. Finally, observations on habitat preferences for each newly described species are provided.

## Introduction

*Archaeopodagrion* is a genus of Neotropical damselflies endemic to the rainforests of the Tropical Andes and the Tumbes-Chocó-Magdalena biodiversity hotspots. This genus has been poorly known; for a long time only the type species *A. bicorne* Kennedy, 1939 and the elusive *A. bilobatum* Kennedy, 1946 were reported from Ecuador, but in the last decade two more species were described: *A. armatum* Tennessen & Johnson, 2010 from southern Ecuador, and *A. fernandoi* Bota-Sierra, 2017 from Colombia, extending the distributional range of the genus almost 700 km north along the Andes.

Here, two new species of *Archaeopodagrion* from localities in Colombia and Ecuador are presented, extending the distribution of the genus further north within the western Andean foothills. Morphological descriptions accompanied with photographs of the diagnostic traits for both species are provided. Additionally, two updated keys for the adults of the known species of *Archaeopodagrion*, one for males (6 spp.) and another one for females (4 spp.) are presented. For *A. recurvatum* sp. nov. the morphological description of the penultimate instar larva is also provided. Besides the morphological descriptions, biological notes, a new distribution map, and some taxonomic remarks are offered, thus adding to the known diversity of this rare genus.

## Materials and methods

Male and female adults and a female larva were collected for *A. recurvatum* but only male adults were collected for *A. mayi*. The F-1 (penultimate instar) larva was preserved in 80% ethanol, the adults were steeped in 96% ethanol for twelve hours and then dried. Specimens are deposited in the ANDES Entomology Museum, Universidad de Los Andes, and the CEUA Entomology Museum, Universidad de Antioquia.

Photographs of morphological structures were taken with a Nikon DS-U3 camera mounted on a Nikon SMZ25 stereomicroscope, and processed with the program NIS elements AR version 4.5. Descriptions were made by examining specimens under a Zeiss Stemi SV6 stereomicroscope, and measurements (in mm) taken with an ocular micrometer and a ruler. Wing nomenclature follows Riek and Kukalová-Peck (1984). Mandible nomenclature follows Watson (1956); labium nomenclature follows Corbet (1953). Wing measurements follow Bechly (1996) and head measurements follow Tennessen (2017). Abbreviations are as follows:

**AL** Abdomen length: maximum length of the abdomen, measured from the first (S1) to the 10^th^ abdominal segment (S10) and including the caudal appendages (cerci and paraprocts), in dorsal view;

**FWL** Forewing (FW) length: maximum length of the forewing, measured from the first cross-vein (ax0) to the furthest point of the wing tip;

**HWL** Hindwing (HW) length: maximum length of the hindwing, measured from the first cross-vein to the furthest point of the wing tip;

**WHW** Hindwing width: maximum width of the hindwing, measured from the nodus to the furthest point of the wing’s posterior edge;

**HFL** Hind femur length: maximum length of the hind femur in lateral view;

**HWd** Head width: maximum width of the head, measured across compound eyes in dorsal view;

**PtL** Pterostigma (Pt) length;

**Px** Postnodal crossveins;

**CL** Cercus length: maximum width of the cercus, measured from the insertion in S10 to the furthest point of the cercus tip in dorsal view;

**S1–10** Abdominal segments;

**TL** Total length: maximum length of the specimen, measured from the furthest edge of the labrum to the 10^th^ abdominal segment (S10) including the caudal appendages (cerci and paraprocts), in dorsal view;

**PpL** Paraproct length: maximum length of the paraprocts, measured from the insertion in S10 to the furthest point of the paraproct tip in dorsal view;

**EpL** Epiproct length: maximum length of the epiproct, measured from the insertion in S10 to the furthest point of the epiproct tip in dorsal view.

### Depositories

**ANDES-E**Entomology collection, Natural History Museum, Universidad de Los Andes, Bogotá, Cundinamarca, Colombia;

**CEUA**Entomology collection, Universidad de Antioquia, Medellín, Antioquia, Colombia.

## Results

### Archaeopodagrion recurvatum sp. nov.

Taxon classificationAnimaliaOdonataMegapodagrionidae

6626B95A-9ABC-5D1E-BB2E-159FE34FFB16

http://zoobank.org/1E28E29E-1352-494F-B77B-6A7DF0E24E44

**Figure 1.**
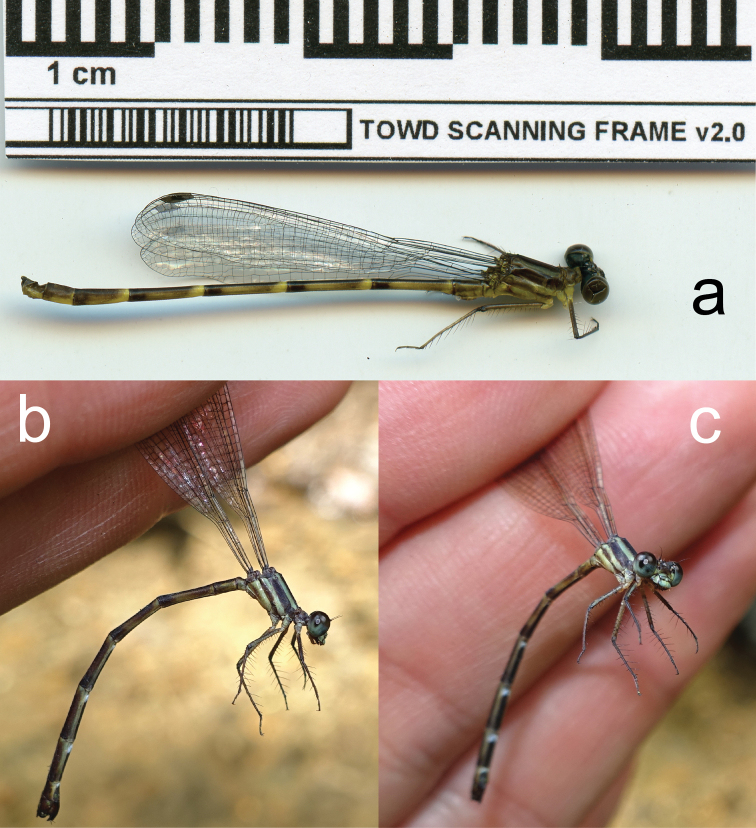
Full body lateral scans and photographs of *Archaeopodagrion recurvatum***A** male holotype **B** and **C** female paratype. Scale bar: 1 cm.

**Figure 2.**
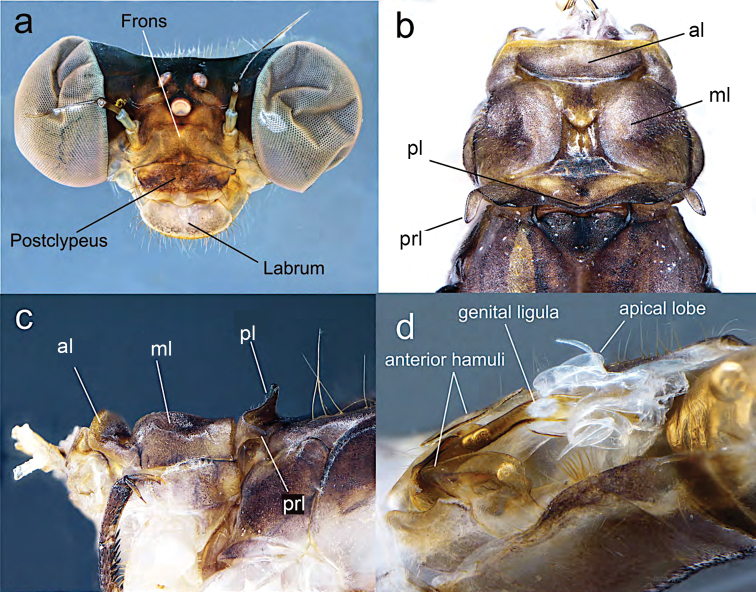
Diagnostic characters of male holotype of *Archaeopodagrion recurvatum***A** head, frontal view **B** pronotum, dorsal view **C** pronotum, lateral view **D** genital ligula. Abbreviations: anterior lobe (al), middle lobe (ml), posterior lobe (pl), pronotal lobe (prl).

**Figure 3.**
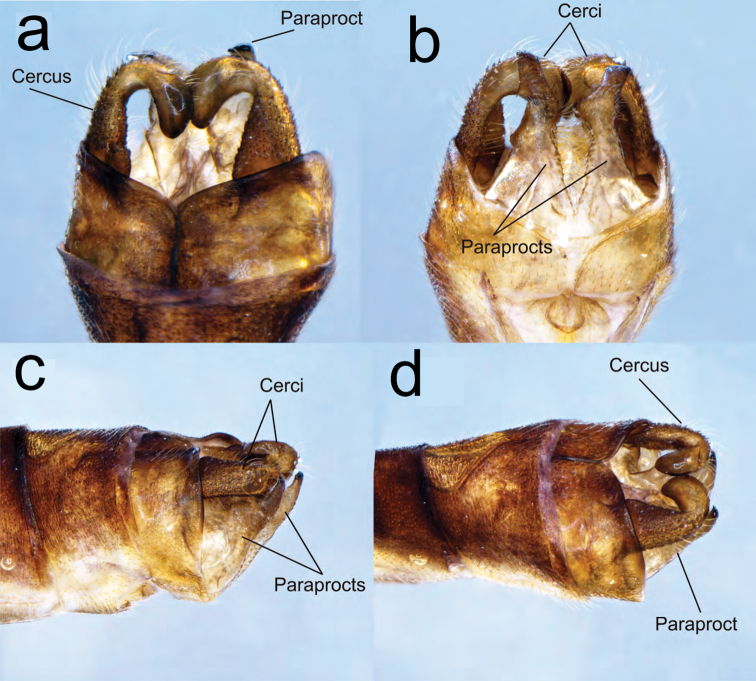
Caudal appendages of teneral male paratype of *Archaeopodagrion recurvatum***A** dorsal view **B** ventral view **C** lateral view **D** dorsolateral view. Caudal appendages might show postmortem distortions.

**Figure 4.**
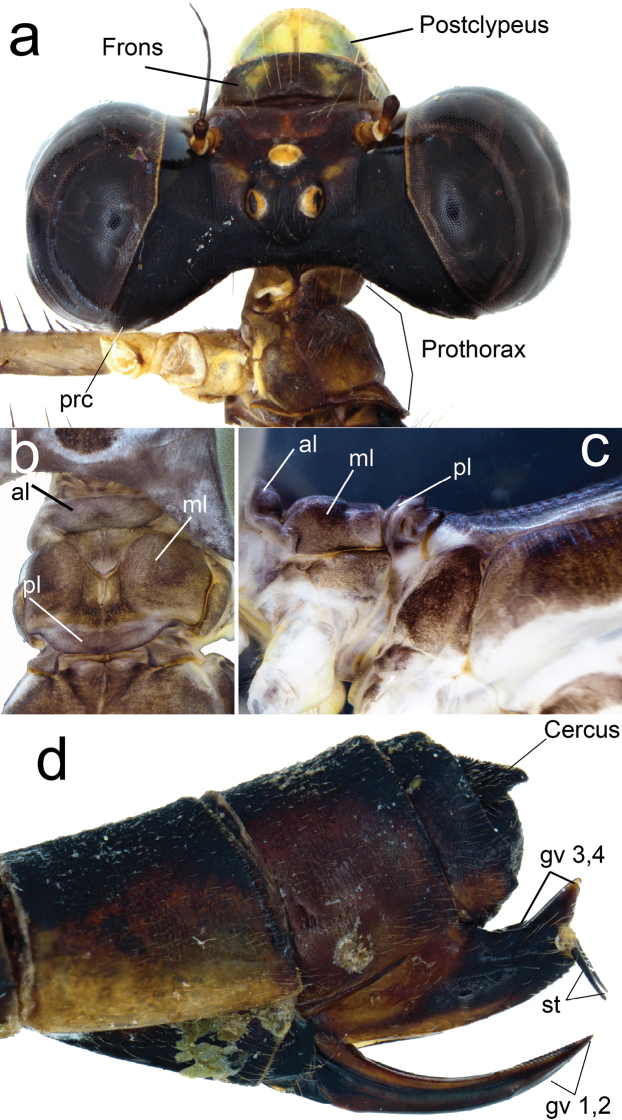
Diagnostic characters of female paratype of *Archaeopodagrion recurvatum***A** head, dorsal view **B** pronotum, dorsal view **C** pronotum, lateral view **D** ovipositor, lateral view. Abbreviations: anterior lobe (al), genital valvae (gv), middle lobe (ml), posterior lobe (pl), paraorbital carina (prc), styli (st).

**Figure 5.**
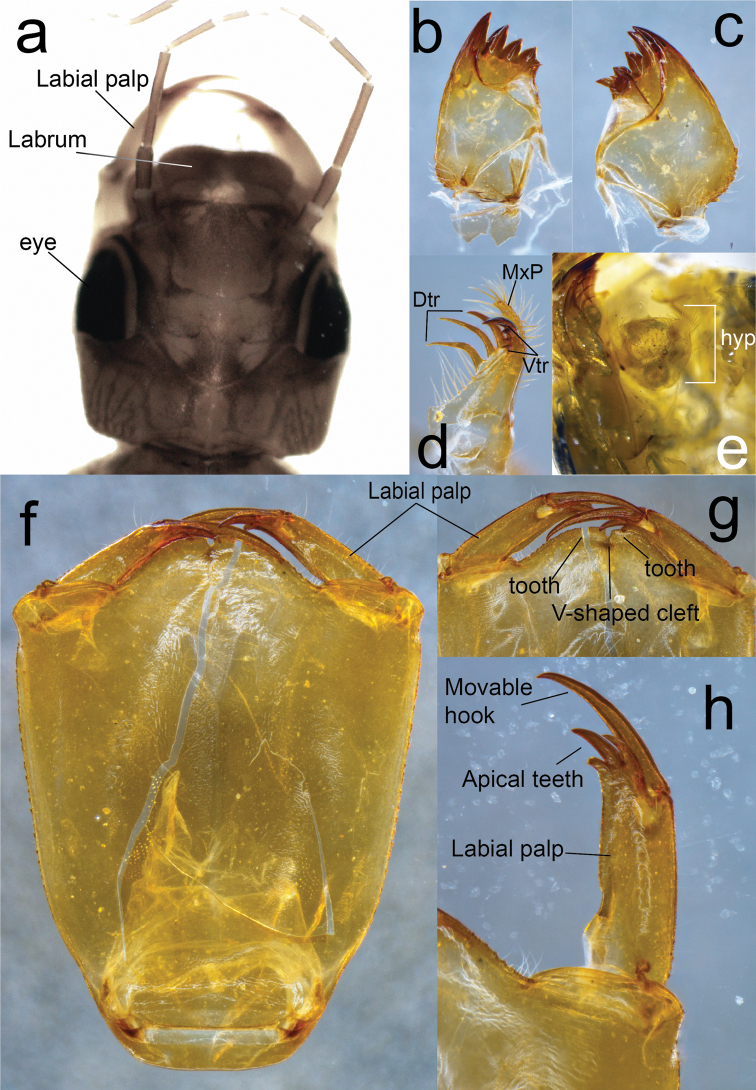
Diagnostic characters of F-1 larva of *Archaeopodagrion recurvatum***A** head, dorsal view **B** right mandible, lateral view **C** left mandible, lateral view **D** galeolacinia, lateral view **E** hypopharynx, dorsal view **F** ligula, ventral view **G** close up of the medial cleft of the ligula, ventral view **H** detail of the labial palp, ventral view. Abbreviations: dorsal teeth (Dtr), hypopharynx (hyp), maxillary palp (MxP), ventral teeth (Vtr).

**Figure 6.**
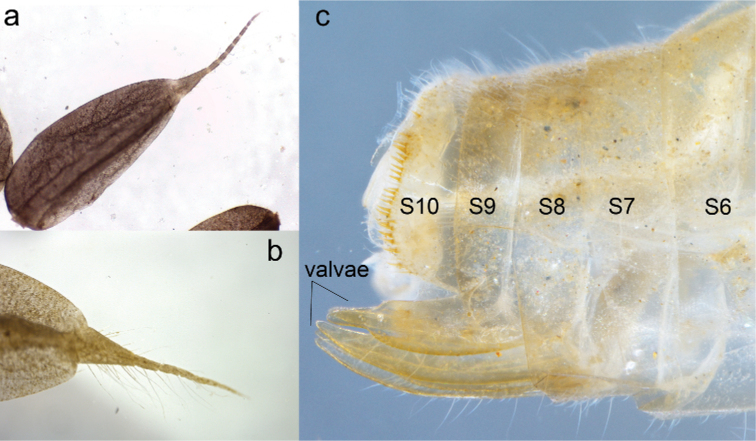
Diagnostic characters of F-1 larva of *Archaeopodagrion recurvatum***A** epiproct, dorsal view **B** paraproct, dorsal view **C** last segments of the abdomen, lateral view.

Figures 1 , 2 , 3 , 4 , 5 , 6

#### Type material.

***Holotype***: 1 adult male, Colombia, Valle del Cauca Department, Dagua Municipality, Farallones Natural National Park, Alto Anchicayá, La Riqueza stream, 3.6094167°N, 76.8845°W, 670 m, taken with aerial net while perched in riparian vegetation, 4 February 2020, VAV and MSH leg. (ANDES-E). ***Paratypes***: 1 male, 1 female as tenerals, same data as holotype; 1 F-1 female larva, same data as holotype, D-net, sand/pebbles bottom in shallow La Riqueza stream bank, but collected 9 July 2016, VAV leg. (ANDES-E). 1 teneral male, 1 adult female, Colombia, Risaralda Department, Santa Cecilia, Alto Amurrupá Reserve, Ranas de Cristal stream, 5.32033°N, 76.17357°W, 620 m, taken with aerial net while perched in riparian vegetation along stream, 31 January 2017, CBJ and JSH leg. (CEUA).

#### Diagnosis.

*Archaeopodagrion recurvatum* lacks the tubercle bearing a hair pencil on the midlength of each paraproct and also lacks a well-developed internal tooth on the cercus, a character present only in *A. bilobatum* and *A. bicorne.* Among the females, *A. recurvatum* differs from the other three females described (females of *A. bilobatum* and *A. mayi* are unknown) by the unique shape of the posterior lobe of the pronotum (Fig. 3B, C). The larvae of *A. recurvatum* lacks the fleshy tubercles or spiniform setae present in the caudal lamellae of *A. fernandoi*; also *A. recurvatum* has a slenderer body than *A. fernandoi* and a different mandibular formula, since the molar crest in the left mandible of *A. recurvatum* possesses twice as many teeth as in *A. fernandoi*. The adults of *A. recurvatum* are also the smallest of the genus (male total length 35.0–38.0 mm, female 34.0 mm), with the average length in the genus ranging from 37.5 m for males and 36.5 mm for females in the smaller known specimens of *A. armatum*, to 45.0 mm for males and 42.0 mm for females in *A. fernandoi* (Kennedy 1946; Tennessen and Johnson 2010; Bota-Sierra 2017).

#### Description.

**Male holotype.** Small-sized damselfly, thorax brown with pale yellow stripes, abdomen brown with pale yellow spots, cerci in dorsal view conspicuously recurved, paraproct tips upturned, with tips contacting distal margin of cerci (Fig. 1A–C). ***Measurements***: holotype: TL 35; AL 28.1; FWL 21.2; HWL 20.9; WHW 2.3; FWPtL 1.1; HWPtL 1.2; HWd 4.6; HFL 4. Risaralda paratype: TL 38; AL 30; FWL 24; HWL 23.1; WHW 2.3; FWPtL 1.2; HWPtL 1.5; HWd 5.1; HFL 4. ***Head***: eyes in life dark gray, with black borders; labium grayish brown, dark brown medially, with long pale setae; labrum and anteclypeus yellow, postclypeus brown, frons grayish brown; vertex, occiput and rear of head black, anterior and postero-dorsal surface of vertex and occiput with sculptured surface that refracts light producing green to red metallic colors; some scattered, dark brown setae on dorsum of head; occiput straight, dark brown with long, brown setae along entire width; antennae dark brown except base, basal half of first antennomere pale yellow (Fig. 2A). ***Thorax***: prothorax light brown. Anterior lobe strongly convex in lateral view, middle and posterior lobes with a deep medial depression, pronotal lobe prolonged laterally into trapezoid processes with a conspicuous spine directed toward mesinfraepisternum (Fig. 2B, C). Middorsal carina of pterothorax brown, mesepisternum brown with a yellowish wide stripe reaching humeral suture. Mesinfraepisternum and mesepimeron brown, metepisternum yellowish along the anterior half, becoming light brown near wing base; metinfraepisternum brown near metepisternum, the posterior half yellowish. Metepimeron yellow, with a slight brown stripe below metathoracic suture. Venter of thorax and coxae yellowish, femora, tibia and claws tan brown. Leg spines 2–4 times longer than spaces between bases of adjacent spines, these spaces increasing distally. Wings hyaline; Px 16–17 in FW, 15–16 in HW; Pt surmounting two to two and a half cells, dark brown with very narrow pale edges next to enclosing veins (Fig. 1A). ***Abdomen***: S1–2 completely light brown; S3–7 light brown dorsally with narrow basal pale-yellow mark connecting to yellow lateral mark and then forming a yellow triangular mark reaching ventral side in S7–8. S9–10 dark brown dorsally, slightly yellow ventrally, S10 with a conspicuous central cleft (Fig. 2A). Genital ligula with two lateroapical recurved flagella as in other species described for the genus (Fig. 2D). Cerci light brown, strongly recurved medio anteriorly with tips touching, giving a heart-shaped appearance, armed with some small denticles along inner margins; paraprocts in lateral view slightly longer than cerci but with divergent acuminate apex strongly recurved dorsally and armed with a small, blunt tooth at the tip (Fig. 3A–D).

**Female paratype. *Measurements***: TL 34; AL 26.8; FWL 22; HWL 21.1; WHW 7.1; FWPtL 1.1; HWPtL 1.2; HWd 5.1; HFL 4. ***Head***: black except: labium pale yellow; labrum mandible base, genae and anteclypeus yellowish blue; postclypeus greyish brown with two yellow spots; antefrons greyish brown, postfrons brown, both with sculptured surface which extends to foramen except for lustrous space between antennae base and eyes, two yellowish blue elongated spots between antenna base and each lateral ocellus; antennae dark brown, base and basal half of first antennomere pale yellow; postocular lobe slightly protruding posteriorly beyond level of hind margin of compound eye, paraorbital carina distinct (Fig. 4A). ***Thorax***: brown with antehumeral and metepisternal yellowish blue stripe, metepimeron pale yellow with a slight brown stripe below metathoracic suture (Fig. 1B, C). Posterior lobe of pronotum convex laterally with a small, angled projection (Fig. 4B, C). Metathoracic legs missing, coxae yellowish blue, femora dark brown externally and yellowish blue internally, tibiae and tarsi brown, armature brown. Eight spurs on the external side of right mesofemur and seven on left mesofemur, each as long as space between them or shorter, gradually increasing in size toward the apex. Eight spurs on the anterior side of right mesotibia and seven on left mesotibia, longer than the spaces between them and gradually decreasing in size toward apex. Tarsal claws with a supplementary tooth. Wings smoky. Pt dark brown surmounting two to two and a half cells, Px 18 in FW and 16 in HW. ***Abdomen***: dark brown except light brown sides of S1–2, apical incomplete rings from S3 to S7 connected to ventrolateral stripes, and S8 ventrolateral stripe. Genital valves brown, internal edges of gv1 and gv2 serrulated, gv3 and gv4 with a large triangular process over the base of long and slender black styli (Fig. 4D).

**Larva.** Penultimate instar F-1, medium size for Zygoptera. Body mostly glabrous, light brown to yellowish; antennae long, light brown. Head large, flattened, abdomen convex dorsally, flattened ventrally. Caudal lamellae saccoid, violaceous, with a long, pale terminal filament. ***Measurements***: TL 13; AL 6; HWd 2; HFL 3; Ep 3.9; Pp 3.5. ***Head***: yellowish brown, almost as wide as long, subhexagonal. Labrum brown, mostly covered by minute spinules, with a large, glabrous, oval, median area and anterior margin widely emarginate medially, with a row of long, white setae as described for *A. fernandoi* (Novelo-Gutiérrez et al. 2020). Clypeus light yellow, with few setae. Frons large, yellowish brown, flat, very finely rugose. Vertex flat, dark brown, with three large white ocelli. Antennae long, 7-segmented, glabrous, all segments yellowish brown, scape barrel-shaped, thicker, pedicel cylindrical, antennomere 3 longest, antennomere 7 shortest, size proportions: 0.30, 0.37, 1.0, 0.55, 0.39, 0.20, 0.10; compound eyes large, not bulging, ventrolateral margin covered with a row of short setae, occiput large, cephalic lobes quadrangular; external margins covered with longitudinal rows of minute spines; occipital margin widely concave (Fig. 5A). Mandibles (Fig. 5B, C) with a movable molar crest, strongly carinate along ventro-lateral margin, baso-lateral margins covered with stout, short spines; formula: R 1+2345 y a (m^1^) b, L1+2345 0 a (m^4^) bd, a > b in both mandibles. Galeolacinia (Fig. 4D) with seven teeth, three dorsal teeth of similar length and robustness, all of them slightly incurved, four ventral teeth with similar sizes and robustness, apical tooth the largest, the basal tooth preceded by a row of approximately six short stiff setae; maxillary palp setose, slightly incurved, ending in a spine shorter than apical tooth of Galeolacinia. Hypopharynx (Fig. 5E) trapezoid, slightly sclerotized, pale yellow, with the distal rounded corners bearing a conspicuous row of long, anteriorly directed white setae; anterior margin slightly rounded, posterior margin slightly concave, shorter. Labium: prementum-postmentum articulation nearly reaching posterior margin of procoxae. Prementum yellow, subrectangular, 0.43 × longer than its widest part, glabrous, shiny, lateral margins finely serrulate, subparallel, gradually converging basally; ligula convex, prominent, distal margin finely serrulated with a short V-shaped median cleft, a minute spine on each side of cleft in dorsal view; labial palp yellow, large, parallel-sided, lateral margin with some short spines on basal third, mesial margin smooth, apical lobe short, ending in three short hooks of different sizes, median hook largest, internal hook shortest; movable hook brown, slightly incurved, sharply-pointed, almost as long as labial palp (Fig. 5F). ***Thorax***: narrower than head, covered with minute setae. Pronotal disc light brown, anterior margin slightly concave, lateral margins strongly produced medially, forming a pronounced, almost acute-angled convexity; posterior margin convex; propleuron spiny, ventral margin widely V-shaped. Pterothorax pale brown with wide, irregular dark-brown spots, ventral margin of mesopleuron serrate. Legs long, yellowish brown, fore legs darker. Femora granulose, tibiae setose. Tarsi pale, with a ventral, longitudinal double row of spiniform setae; tarsal claws simple, all with pulvilliform empodium. Anterior and posterior wing sheaths slightly divergent, reaching the basal half and posterior margin of S4, respectively. ***Abdomen***: convex dorsally, flat ventrally, slightly narrowing posteriorly. Tergum including posterior margins of S1–10 light brown, covered with minute white setae. Sternum bare, creamy pale. S3–5 distorted, the female gonapophyses surpassing well beyond posterior margin of S10, lateral valvae creamy-pale, ventrally setose, central valvae yellow, smooth, longer, all roundly pointed (Fig. 6C), as in *A. fernandoi* (Novelo-Gutiérrez et al. 2020). Cerci not visible. Caudal lamellae pale violet, saccoid, with abundant, long white setae on dorsum, with a long pale, setose, gradually tapering terminal filament. Epiproct (Fig. 6A) lacking fleshy tubercles or spiniform setae along midline; terminal filament divided into 7 segments, almost 1/3 shorter than the saccoid portion length of epiproct. Paraproct (Fig. 6B) without fleshy tubercles or spiniform setae; terminal filament shorter than saccoid portion length of epiproct, divided into seven segments covered with long, white setae. S8–9 bearing genital valvae (Fig. 6C).

#### Distribution.

**Figure 7.**
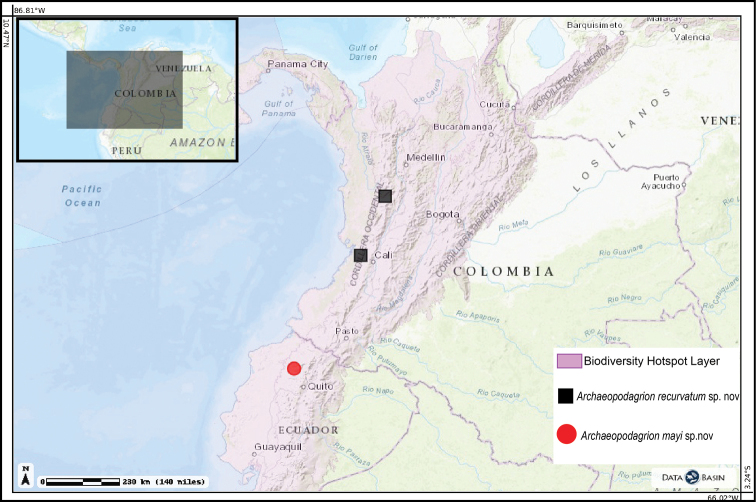
Distribution map for the new species of *Archaeopodagrion*. Black dots represent type localities.

Known only from the holotype and paratype localities on the western slope foothills of the West Andean Cordillera at the Colombian departments of Risaralda and Valle del Cauca (Fig. 7).

#### Etymology.

Named *recurvatum* (from the Latin *recurvatum*: curved inwards, with tips that are directed back to the point of origin) due to the peculiar structure of the male’s cerci.

### Archaeopodagrion mayi sp. nov.

Taxon classificationAnimaliaOdonataMegapodagrionidae

692EBE82-245C-59FA-BB25-DFD9006A7DB9

http://zoobank.org/65471F12-C179-47E5-8114-9514E5458214

Figures 8 , 9 , 10

#### Type material.

***Holotype***: 1 adult male, and 1 adult male ***paratype***, both Ecuador, Imbabura Province, Reserva Natural Los Cedros, near Cascada Vieja Trail, 0.308986°N, 78.779328°W, 500 m, taken with aerial net while perched in lianas hanging from a small stream canyon wall, 4 December 2013, MSH leg. (ANDES-E).

#### Diagnosis.

The males of *Archaeopodagrion mayi* are medium-sized, with total length ranging from 40.0 to 43.0 mm. They present a tubercle bearing a hair pencil on the midlength of each paraproct, a character shared with the males of *A. armatum* and *A. fernandoi*, and lack a well-developed internal tooth on cercus, a character shared only with *A. recurvatum*.

#### Description.

**Figure 8.**
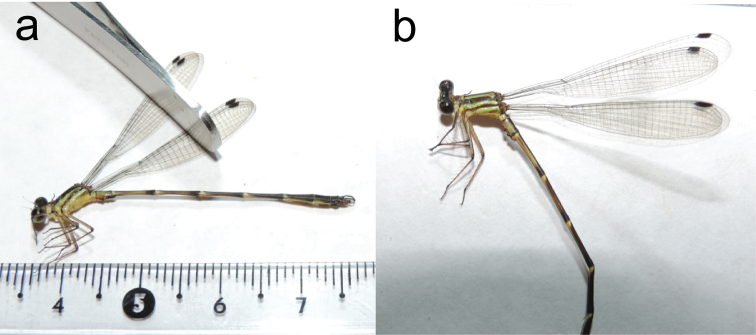
Full body lateral photographs of *Archaeopodagrion mayi***A** male holotype **B** male holotype head and thorax closeup. Scale bar: 1 cm.

**Figure 9.**
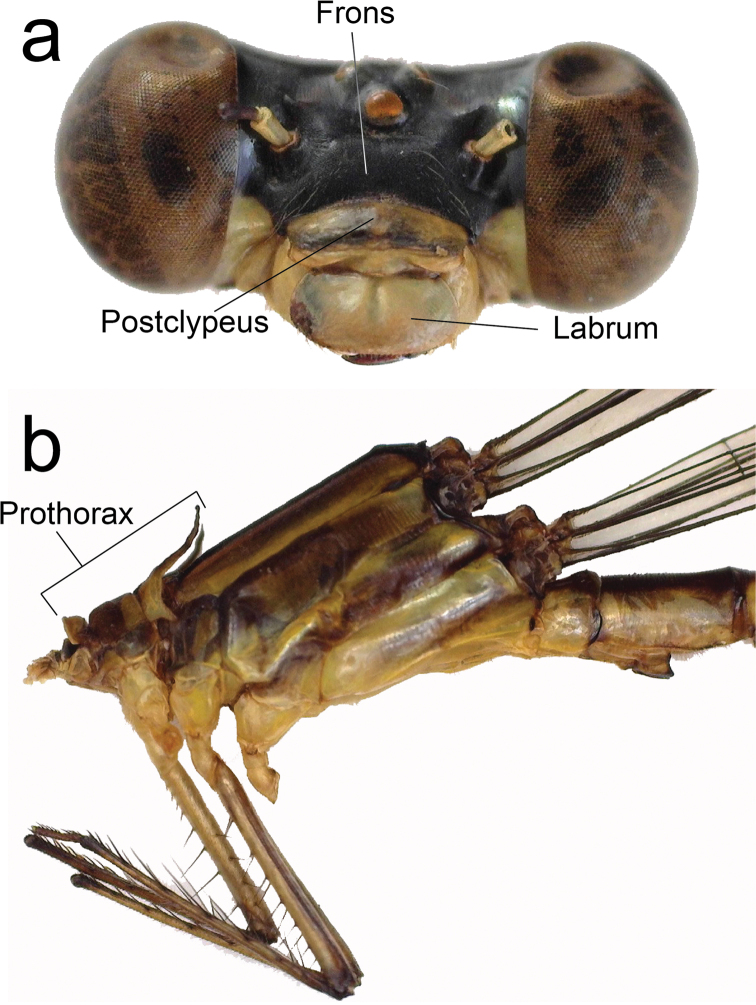
Diagnostic characters of male holotype of *Archaeopodagrion mayi***A** head, frontal view **B** thorax, lateral view.

**Figure 10.**
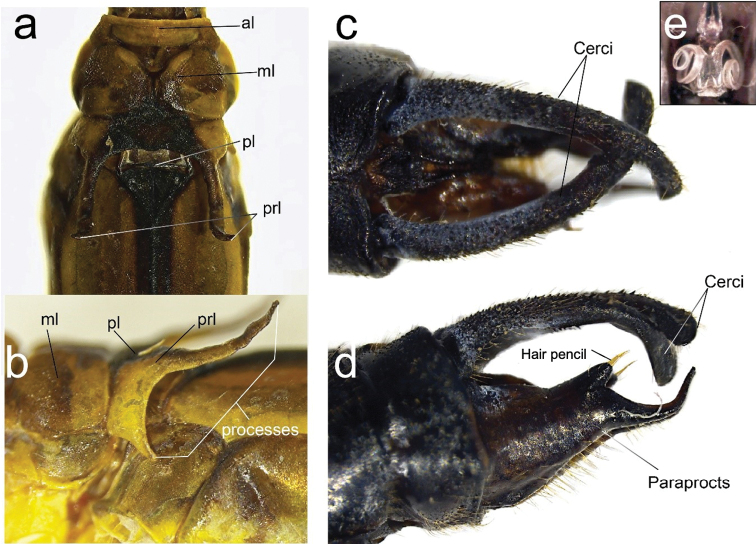
Diagnostic characters of male holotype of *Archaeopodagrion mayi***A** pronotum, dorsal view **B** pronotum, lateral view **C** caudal appendages, dorsal view **D** caudal appendages, lateral view **E** genital ligula, ventral view. Caudal appendages might show postmortem distortions. Abbreviations: anterior lobe (al), middle lobe (ml), posterior lobe (pl), pronotal lobe (prl).

**Male holotype.** Medium-sized damselfly, thorax brown with greenish yellow stripes, abdomen dark brown dorsally and light brown ventrally, with greenish yellow pale spots (Fig. 8). Abdominal appendages with characteristic morphology, comprising cylindrical cerci with tips directed posteroventrally, longer than paraprocts, and paraprocts with tips acute and directed dorsally (Fig. 10A–D). ***Measurements***: holotype: TL 40; AL 34.1; FWL 26.5; HWL 25; WHW 2.5; FWPtL 1.2; HWPtL 1.5; HWd 4.1; HFL 4; CL 2. Paratype: TL 43; AL 36; FWL 26.5; HWL 25; WHW 2.3; FWPtL 1; HWPtL 1.3; HWd 4.1; HFL 4; CL 1.8. ***Head***: eyes brown; head black except: labium, labrum mandible base, genae, and anteclypeus pale greenish yellow; labium and labrum with long, pale setae; postclypeus greyish yellow, antefrons and postfrons dark brown, both with sculptured surface except lustrous space between antennae base and eyes and extending to occipital foramen; antennae dark-brown except pale yellow base, postocular lobe slightly protruding posteriorly beyond level of hind margin of compound eye, paraorbital carina distinct (Fig. 9A). ***Thorax***: prothorax bright yellow with brown spots, pterothorax brown with antehumeral and metepisternal yellow stripe, metepimeron brown with a yellow stripe below metathoracic suture (Figs 8B, 9B). Anterior lobe of the prothorax subquadrangular, middle lobes arcuate medially (Fig. 10A), the posterior lobe of pronotum convex, prolonged laterally into two pairs of processes: one on superior margin and the other on inferior margin; superior processes long, curved dorsoposteriorly, their tips blunt and extending over a third of the pterothorax; inferior processes shorter, less than half the length of superior processes, and extending posteroventrally (Fig. 10B). Venter of thorax and coxae yellow, femora dark brown externally and pale brown internally, tibiae, tarsi and armature brown. Leg spines 2–3 times longer than spaces between bases of adjacent spines, interval between spurs subequal along all legs. Tarsal claws with supplementary tooth. Wings hyaline. Pt dark brown surmounting two to two and a half cells, ratio between distal and proximal length approximately 1:1. Px 18 in FW and 16 in HW. ***Abdomen***: dark brown, S1 and S2 with yellow lateral stripes, S3–7 dark brown dorsally with narrow basal yellow ring connecting laterally to yellow stripe along ventral side in S7 and S8. S9 and S10 dark brown (Fig. 8). Cercus cylindrical, covered with denticles and scattered short setae, in lateral view longer than paraproct (Fig. 10C, D); paraprocts in lateral view with a strong ventral tubercle located at midlength, the tubercle bearing a conspicuous hair pencil apically, tips of paraprocts recurved, acute, hook shaped (Fig. 10D). Genital ligula with two lateroapical recurved flagella as in the other species described for the genus.

#### Distribution.

Known only from the holotype and paratype locality in the western foothills of the Ecuadorian Andes in Imbabura Province (Fig. 7).

#### Etymology.

Named *mayi*, an adjective in the genitive case, after Dr. Michael L. May for his great contributions on the study of odonates, whose work ranges from basic taxonomy and systematics to complex ecological and physiological questions related to migratory patterns and to the body temperature regulation of dragonflies. Dr. May has established the foundation for many research topics in Odonatology and has supported the development of several odonatologists and their research.

##### Biological observations

The specimens of *A. recurvatum* were collected at two different locations with similar characteristics along the Colombian Western Andean foothills, where the climate is tropical, with temperatures ranging from 22 °C to 27 °C, high precipitation (> 3000 mm per year [Pravettoni 2019]) and tropical rainforest as the predominant vegetation. They were found on small to medium-sized fast flowing streams, with sand and gravel substrates surrounded by a mix of boulders and exposed bedrocks, interspersed with waterfalls, pools and rapids, within primary and undisturbed forests. The waters were clear, clean, and highly oxygenated. *Archaeopodagrion recurvatum* coexisted there with damselflies of other families such as Heteragrionidae, Polythoridae, and Platystictidae. Those streams are highly dependent on precipitation: during the wet season, the stream water level can increase 0.50 to 1.0 m, decreasing in the same proportion during the dry season. When the waters recede, apparently there are more available spaces for adults to emerge, using semi-submerged large stones and sticks as attachment substrate. Amaya-Vallejo (2009) identified odonate assemblages associated with different habitats within the Anchicayá zone, thus classifying most of the species inhabiting the previously described streams as stenoecious, or able to live only in a restricted range of habitats.

*Archaeopodagrion mayi* was collected at Los Cedros Natural Reserve in Ecuador. This reserve is located in the southernmost area of the Cordillera de la Plata, lying on the western side of the Andes mountains. The temperature generally fluctuates between 16–25 °C and the humidity can be as high as 100% due to the high level of precipitation (> 3000 mm per year [Pravettoni 2019]). The dominant type of vegetation is tropical rainforest. The specimens were found in a swampy trail left by a stream located inside a small canyon; the walls were covered with long roots and lianas. Both males were perching at the end of these roots, with their wings open as in individuals of *Heteragrion* and somewhat resembling females of this genus. The collection trip occurred right after the dry season and the climatic conditions were similar to the ones in the Colombian locations. The stream water level may have been reduced and *A. mayi* may have the same emergence habits as *A. recurvatum*. Polythoridae and Heteragrionidae were collected at the same place and considering the characteristics of the habitat and the classification made by Amaya-Vallejo (2009), *A. mayi* may also be a stenoecious species thus sharing similar habitat requirements with *A. recurvatum*. Despite the habitat requirements for both species, due to lack of surveys we suggest these to be data deficient category for the IUCN red species list.

##### Taxonomic remarks

The phylogenetic position of *Archaeopodagrion* was expected to be close to the Malagasy genus *Tatocnemis* based on morphological characteristics (Rácenis 1959). Dijkstra et al. (2014) proposed the family Philogeniidae for *Archaeopodagrion* and *Philogenia* genera based on molecular evidence only, but morphologically, the only character shared by them is the coiled flagella on genital ligula, with no further evidence of other reliable diagnostic characters (Bota-Sierra 2017). With the description of the larva of *Archaeopodagrion* (Novelo-Gutiérrez et al. 2020), some important taxonomic information supported Philogeniidae as a monophyletic group including the shape of the antennae and the caudal lamellae, the structure of the galeolacinia of the maxilla, the maxillary palp and the distointernal margins of tibiae and the prementum. Autapomorphic characters separating larvae of *Archaeopodagrion* from those of *Philogenia* become less apparent with the description of *A. recurvatum* because the caudal lamellae with basal, spiny, fleshy tubercles present in *A. fernandoi* are lacking in *A. recurvatum*. We propose that the mandibular formula is the most reliable character for separating larvae of *Archaeopodagrion* and *Philogenia*, following Novelo-Gutiérrez et al. (2020): the molar lobe in the right mandible of *Archaeopodagrion* does not present m denticles but it does in *Philogenia*, the molar lobe in the left mandible of *Archaeopodagrion* has only two m denticles but the number varies in *Philogenia*, and the tooth d in the left mandible of *Archaeopodagrion* is small, bluntly-pointed and close to tooth b whereas in *Philogenia* the shape is different and its position is quite variable.

### Updated key for the adults of *Archaeopodagrion* (modified from Bota-Sierra 2017)

#### Key to males

**Table:** 

1	Paraprocts with a tubercle bearing a hair pencil (Fig. 10D)	**2**
–	Paraprocts lacking a tubercle bearing a hair pencil	**4**
2	Paraproct apex with two pointed processes	***A. armatum***
–	Paraproct apex ending in a single process (Fig. 10C)	**3**
3	Tips of paraprocts slightly recurved, shorter in length compared to cercus in lateral view	***A. fernandoi***
–	Tips of paraprocts strongly recurved, paraproct subequal in length compared to cercus in lateral view (Fig. 10D)	***A. mayi***
4	Cerci with a sharp tooth internally	**5**
–	Cercus lacking an internal sharp tooth (Fig. 3A–D)	***A. recurvatum***
5	Cercus with an internal sharp tooth at midlength; cercus slightly curved in lateral view	***A. bicorne***
–	Cercus with an internal sharp tooth at basal 1/4; cercus strongly curved in lateral view	***A. bilobatum***

#### Key to females

Note: the females of *A. bilobatum* and *A. mayi* are unknown.

**Table:** 

1	Posterior lobe of pronotum convex or with projections at posterolateral corners	**2**
–	Posterior lobe of pronotum laterally with a large, arched, hollowed out recurved quadrate process	***A. armatum***
2	Posterior lobe of pronotum convex with lateral small angled projections (Fig. 4B, C)	***A. recurvatum***
–	Posterior lobe of pronotum with posterolateral projections blunt or rounded	**3**
3	Posterolateral projections on posterior lobe of pronotum acute	***A. fernandoi***
–	Posterolateral projections on posterior lobe of pronotum wide and rounded	***A. bicorne***

## Supplementary Material

XML Treatment for Archaeopodagrion recurvatum

XML Treatment for Archaeopodagrion mayi
